# Breath, beyond blood: the role of gaseous biofluids in medical research

**DOI:** 10.4103/mgr.MEDGASRES-D-26-00055

**Published:** 2026-07-03

**Authors:** Susmita Roy, Ronald Sroka, Kiran Sankar Maiti

**Affiliations:** Laser-Forschungslabor, LIFE-Center, LMU University Hospital, LMU Munich, Planegg, Germany; Department of Urology, LMU University Hospital, LMU Munich, Munich, Germany; TUM School of Natural Sciences, Department of Chemistry, Technical University of Munich, Garching, Germany

##  

Thousands of parallel and sequential biochemical reactions occur within living cells, generating a diverse array of metabolites as byproducts. Systematic analysis of these metabolites offers insight into the underlying biochemical processes and, collectively, provides a comprehensive view of the body’s physiological state.^1^ Metabolite analysis of blood has long been an integral part of clinical diagnostics and health monitoring. Despite its widespread use, current blood-based assays remain effective for only a limited subset of diseases in which metabolite compositions undergo pronounced changes.^2^ Elevated blood glucose levels indicative of diabetes, reduced hemoglobin levels associated with anemia, and abnormal bilirubin concentrations reflecting impaired liver function. While these biomarkers enable robust diagnosis, they represent cases in which disease-related biochemical alterations are large and readily detectable by biochemical analysis.

In contrast, many chronic and life-threatening conditions, including cancer, Parkinson’s disease, and heart failure, progress silently over extended periods. For these diseases, diagnosis often occurs only after clinical symptoms emerge, at which point therapeutic options are frequently limited. This diagnostic delay highlights a fundamental limitation of current blood analysis strategies: their insufficient sensitivity and selectivity to detect the subtle biochemical perturbations that characterize early disease states.

Blood is an exceptionally complex molecular matrix, containing thousands of metabolites spanning a wide dynamic range of concentrations.^3^ Identifying and accurately quantifying disease-responsive metabolites (biomarkers) within this background remains a major analytical challenge, particularly when early pathological changes result in only marginal concentration shifts. Although substantial efforts have been devoted to the development of advanced analytical tools aimed at detecting these minute variations, existing approaches have yet to deliver consistently convincing outcomes for early-stage disease detection. Among many, laser spectroscopy-based vibrational information of targeted molecules holds a lot of promise for rapid biomarker identification. A large number of research groups are taking their first steps toward the identification and quantification of biomarkers with subtle variations.^2^ However, overlapping spectral bands, inadequate spectral decomposition methods, and a lack of understanding of human metabolic characteristics still limit their application in clinical practice.

Although gaseous metabolite analysis has only recently gained prominence as a diagnostic modality, its conceptual foundations trace back to antiquity. Early physicians, including Hippocrates, Galen, and Avicenna, recognized the clinical significance of odor and systematically integrated olfactory assessment into medical evaluation.^3^ Distinctive scent signatures were linked to specific pathologies; for example, a sweet body odor was associated with diabetes, whereas a pungent ammonia-like odor suggested renal dysfunction.

In contemporary research, renewed interest in disease-associated volatile organic compounds (VOCs) has further underscored the diagnostic potential of olfaction. Notably, a “super smeller” demonstrated the remarkable ability to detect Parkinson’s disease based on odor.^4^ Similarly, trained animals have exhibited the capacity to identify prostate cancer through urine scent detection. Despite these compelling observations, both human and animal olfactory systems remain inherently limited in sensitivity, specificity, and scalability, rendering them insufficient for the reliable detection of the wide spectrum of diseases that burden modern healthcare systems.

Significant advances in gas chromatography–mass spectrometry-based VOC analysis, pioneered in part by two-time Nobel laureate Linus Pauling in the 1970s, have markedly enhanced the analytical depth, sensitivity, and molecular resolution of VOC profiling. Despite its outstanding sensitivity and molecular specificity, mass spectrometry has not yet been widely adopted as a routine clinical diagnostic tool. This limitation arises from the complexity of analytical workflows, high operational costs, large instrument footprint, and challenges in translating analytical performance into robust disease-specific diagnostics.^5^

Electronic nose technologies offer an attractive alternative in terms of compactness and cost-effectiveness. However, their limited chemical specificity and difficulties in achieving reproducible, clinically validated diagnostic performance have hindered their clinical acceptance. Quartz-enhanced photoacoustic spectroscopy demonstrates exceptional detection sensitivity for trace gases. Nevertheless, its intrinsically narrow spectral interrogation window restricts simultaneous multi-species analysis, rendering it more suitable for targeted detection once specific biomarkers have been definitively identified. Although the detection sensitivity of Fourier-transform infrared spectroscopy is generally lower than that of gas chromatography–mass spectrometry or quartz-enhanced photoacoustic spectroscopy, its inherently broadband spectral coverage provides a powerful and comprehensive analytical platform for biomarker discovery.^1^ This broad spectral capability enables simultaneous, multi-component analysis, making Fourier-transform infrared particularly advantageous for exploratory investigations aimed at identifying novel disease-associated VOC signatures.^6^

The advancement of infrared spectroscopy–based VOC identification has been enabled by the breakthrough development of an effective water vapor suppression technique for gaseous biofluids.^7^ In this approach, the sample is passed at a controlled flow rate through a copper spiral maintained in a cryogenic bath at –60°C. Owing to the high vapor pressure of water, a substantial fraction of water vapor condenses and freezes within the copper tubing, resulting in a nearly water-free biofluid reaching the measurement cell. Near-complete removal of water vapor eliminates the dominant “umbrella effect” of water vapor, thereby unmasking the intrinsic molecular fingerprints of trace metabolites, as illustrated in **Figure 1**. Coupled with non-destructive data acquisition, advanced spectral deconvolution, rigorous statistical modeling and multi-metabolite profiling offers a promising pathway toward an accurate diagnostic platform with clinically acceptable sensitivity and specificity.^8^

**Figure 1:**
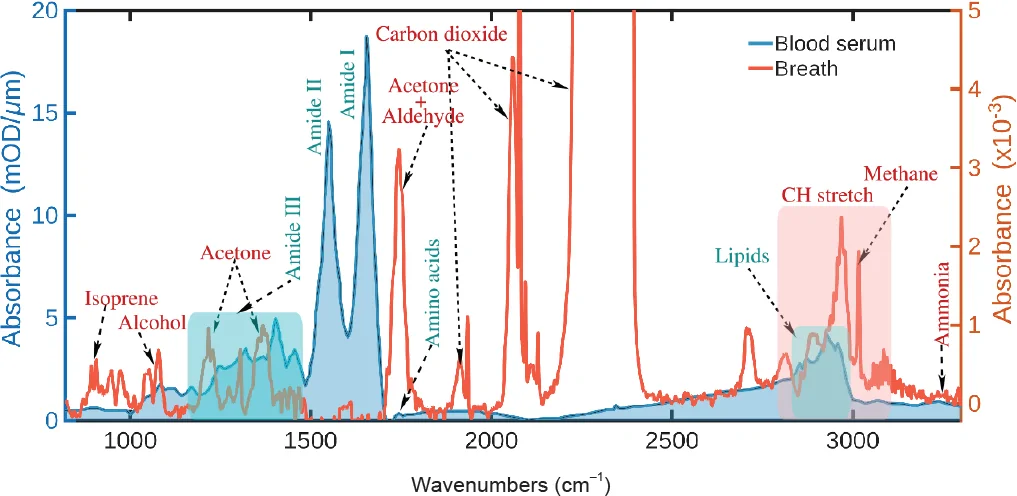
Typical infrared absorption spectra of blood serum and exhaled breath. The left-hand y-axis corresponds to the absorption intensity of blood serum, while the right-hand y-axis represents the absorption intensity of the breath spectrum. Amide III and lipid bands in the serum spectrum are highlighted with a cyan shaded box, whereas the CH stretching modes in the breath spectrum are highlighted with a red shaded box. Spectroscopic data were acquired and preprocessed using OPUS 7.8 software, and the final plots were generated using Matlab R2025b. mOD: Milli-optical density.

This technique has been successfully applied to identify candidate biomarkers for prostate cancer, cerebral palsy, and bacterial infections, thereby demonstrating its potential as a powerful platform for early and pre-symptomatic disease detection.^6^,^9^ Gas–phase biofluid-based diagnostics offer several compelling advantages over conventional blood-based approaches. Although venipuncture is widely regarded as minimally invasive and routine, it nonetheless entails physical discomfort and may induce psychological stress. These limitations are particularly consequential for vulnerable populations, including pediatric patients, in whom blood collection can be technically challenging, clinically contraindicated, or ethically sensitive.^9^ Furthermore, by monitoring the temporal evolution of metabolite concentrations, this technique has the potential to serve as a valuable diagnostic tool for determining disease states. To date, it has not been applied directly for disease state evaluation; however, it has demonstrated utility in assessing metabolite changes during recovery states.^1^,^10^

In contrast, gaseous biofluids–skin gas emissions^11^ exhaled breath and urine headspace, can be collected entirely noninvasively, without imposing physical or psychological burden. The simplicity and patient-friendly nature of sample collection make gas-phase diagnostics especially attractive for large-scale screening and longitudinal monitoring, such as assessing treatment response during follow-up or detecting disease recurrence. Moreover, gaseous biofluids typically require minimal sample preparation compared with blood, enabling rapid turnaround and repeated measurements. For instance, infrared spectroscopic analysis of gaseous biofluids can be completed within approximately 25 minutes, encompassing sample collection, preparation, and measurement,^8^ whereas blood-based analyses often require several hours.^12^ An in-built sensor-based smart mask is emerging as a promising alternative for VOC sensing, capable of providing real-time information on VOC levels. However, the technology is currently limited to detecting a few gases at relatively high concentrations and is not yet suitable for trace-level analysis.

From an analytical perspective, gas-phase infrared spectroscopy provides distinct molecular fingerprints characterized by narrow and well-resolved spectral features. In contrast, liquid-phase infrared spectra frequently consist of overlapping bands (**Figure 1**) arising from numerous molecular species, complicating unambiguous biomarker identification, particularly during early-stage disease progression, when alterations in biomarker composition are subtle and difficult to resolve. The reduced spectral congestion in the gas phase facilitates more straightforward molecular discrimination and enhances analytical specificity. For instance, breath-based infrared spectroscopy has already demonstrated more than 90% accuracy in distinguishing prostate cancer patients from a healthy cohort, whereas blood serum-based laser spectroscopy achieves only around 80% accuracy.^6^ Collectively, these advantages position gaseous biofluid analysis as a promising and potentially transformative diagnostic modality for the early detection and monitoring of life-threatening diseases.

*The authors declare that no generative AI was used in the preparation of this manuscript.*
